# Baseline immunophenotypic profile of bone marrow leukemia cells in acute myeloid leukemia with nucleophosmin-1 gene mutation: a EuroFlow study

**DOI:** 10.1038/s41408-023-00909-4

**Published:** 2023-09-04

**Authors:** Sergio Matarraz, Pilar Leoz, Ana Yeguas-Bermejo, Vincent van der Velden, Anne E. Bras, Jose I. Sánchez Gallego, Quentin Lecrevisse, Rosa Ayala-Bueno, Cristina Teodosio, Ignacio Criado, María González-González, Juan Flores-Montero, Alejandro Avendaño, María B. Vidriales, María C. Chillón, Teresa González, Ramón García-Sanz, María I. Prieto Conde, Neus Villamor, Laura Magnano, Enrique Colado, Paula Fernández, Edwin Sonneveld, Jan Philippé, Michaela Reiterová, Juan C. Caballero Berrocal, Francisco J. Diaz-Gálvez, Fernando Ramos, Julio Dávila Valls, Raquel Manjón Sánchez, Jackeline Solano Tovar, Xavier Calvo, Luis García Alonso, Leonor Arenillas, Sara Alonso, Ariana Fonseca, Covadonga Quirós Caso, Jacques J. M. van Dongen, Alberto Orfao

**Affiliations:** 1 Translational and Clinical Research Program, Centro de Investigación del Cáncer (IBMCC; CSIC-University of Salamanca); Cytometry Service, NUCLEUS; Department of Medicine, University of Salamanca (USAL) and Institute of Biomedical Research of Salamanca (IBSAL), Salamanca, Spain; 2 Biomedical Research Networking Centre Consortium of Oncology (CIBERONC), Instituto de Salud Carlos III, 28029 Madrid, Spain; 3 Hematology Department, University Hospital of Salamanca, CIBERONC (CB16/12/00233), IBSAL, Accelerator program and Centro de Investigación del Cáncer (IBMCC; CSIC-University of Salamanca), Salamanca, Spain; 4 Department of Immunology, Erasmus MC, University Medical Center Rotterdam, Rotterdam, The Netherlands; 5 Hematology Service, Hospital Clinic, Barcelona, Spain; 6 Hematology Department and Laboratory Medicine Department, Hospital Universitario Central de Asturias, Oviedo, Spain; 7 FACS/Stem Cell Laboratory, Kantonsspital Aarau, Aarau, Switzerland; 8 Dutch Childhood Oncology Group, The Hague, The Netherlands; 9 Department of Diagnostic Sciences, Faculty of Medicine and Health Sciences, Ghent University, Ghent, Belgium; 10 CLIP-Department of Pediatric Hematology and Oncology, Second Medical Faculty, Charles University and University Hospital Motol, Prague, Czech Republic; 11 Department of Hematology, Hospital Clínico Universitario de Valladolid, Valladolid, Spain; 12 Department of Hematology, Hospital Universitario de Burgos, Burgos, Spain; 13 Department of Hematology, Complejo Asistencial Universitario de León, León, Spain; 14 Department of Hematology, Complejo Asistencial de Ávila, Ávila, Spain; 15 Department of Hematology, Complejo Asistencial de Zamora, Zamora, Spain; 16 Department of Hematology, Complejo Asistencial Universitario de Palencia, Palencia, Spain; 17 Pathology Service, Hospital del Mar, Barcelona, Spain; 18 Hematology Service, University Hospital of Getafe, Madrid, Spain; 19 Department of Immunology, Leiden University Medical Center, Leiden, The Netherlands

**Keywords:** Acute myeloid leukaemia, Leukopoiesis

Dear Editor,

Molecular techniques are the gold standard method for the diagnosis of AML with mutated nucleophosmin gene (*NPM1*^*mut*^). However, their worldwide availability is limited and they provide limited insight into disease heterogeneity. Hence, surrogate markers of *NPM1*^mut^ are used for fast diagnostic screening of the disease [1], including, among others, immunohistochemical detection of cytoplasmic NPM1 (NPM1c) [2], cup-like nuclear morphology [3], normal karyotype, and/or recurrent flow cytometry profiles -e.g., CD34 negativity, and/or a phenotype resembling acute promyelocytic leukemia (APL)- [4]. Nevertheless, some of these methods are also not widely available, they show limited sensitivity (e.g., low or absent NPM1c expression, particularly among monoblastic/monocytic AML-*NPM1*^mut^) [5], frequently lack standardized procedures [1], and they might also bring limited information about disease heterogeneity.

AML-*NPM1*^mut^ leukemia cells present with heterogeneous cytomorphology and immunophenotypic patterns of lineage commitment and antigen expression, including phenotypes associated with *FLT3*-internal tandem duplication (ITD) and a poor outcome [6]. In parallel, neutrophil-lineage commitment of AML-*NPM1*^mut^ cells has been linked with underlying *TET2* and *IDH1/2* mutations, while the presence of monocytic lineage-committed and immature *NPM1*^mut^ cells has been related to *DNMT3A* mutations; indeed, these three patient subgroups show increasingly worse outcomes [4]. Strikingly, *NPM1*^mut^*FLT3*-ITD^−^ patients displaying immature immunophenotypes show strong similarities with *NPM1*^mut^*FLT3*-ITD^+^ cases regarding leukemia cell transcriptomic profiles, response to therapy and poorer outcomes [7]. Thus, baseline flow cytometric characterization of AML-*NPM1*^mut^ leukemia cell heterogeneity might contribute to guiding treatment decisions in these patients. Despite the above associations, specific immunophenotypic patterns of AML-*NPM1*^mut^ remain to be fully defined.

Herewith, we performed a detailed flow cytometric characterization of different subsets of BM leukemia cells from 377 AML patients, including 201 AML-*NPM1*^mut^, 144 AML-*NPM1*^wt^ and 32 APL patients, based on the EuroFlow 8-color acute leukemia orientation tube (ALOT) and the AML/MDS antibody panel (Supplementary methods and Supplementary Table 1). *FLT3*-ITD was detected in 33% of AML-*NPM1*^mut^ cases, 19% of AML-*NPM1*^*wt*^ and 34% of APL patients. Our aim was to identify reliable phenotypic profiles for fast screening of *NPM1*^mut^ and/or *FLT3-*ITD to guide subsequent molecular diagnostic approaches that can be applied worldwide and provide a better understanding of disease heterogeneity.

Our data confirm that AML-*NPM1*^mut^ patients usually present with high BM leukemia cell percentages at similar levels to APL (Supplementary Table 2). However, *NPM1*^mut^ cells displayed highly heterogeneous immunophenotypes, consisting of three main BM cell populations: (1) immature leukemia cells showing stem cell-like features (i.e., CD117^+^HLADR^+^, 46% of cases; (2) neutrophil lineage-committed CD117^+/het^ HLA-DR^−^ (45%), and/or; (3) monocytic-lineage AML cells expressing CD64^+/hi^ HLA-DR^+^ and variable CD117 levels (54% of cases) (Supplementary Fig. 1). The differential immunophenotypes observed for these AML cell populations in AML-*NPM1*^mut^ vs. AML-*NPM1*^wt^ are detailed in Supplementary Results and Supplementary Table 3. Noteworthy, the relative distribution of AML cell populations defined seven distinct immunophenotypic patterns: (1) a predominant expansion of one (of the above three) leukemia cell population (≥80% of total BM leukemia cells; *n* = 3 profiles), and; (2) mixed expansions of >1 leukemia cell population (each representing ≥20% of all BM AML cells; *n* = 4 patterns) (Supplementary Fig. 1). The AML-*NPM1*^mut^ patients from the former group more frequently showed predominant expansions of neutrophil- (28% of cases), followed by monocytic-lineage (19%) and immature leukemia cells (13%). Conversely, mixed leukemia cell expansions included mixed (1) immature and monocytic (23%), (2) monocytic and neutrophil (7%), (3) immature and neutrophil (5%) and (4) immature plus neutrophil- and monocytic-lineage AML cells (5% of cases).

The distribution of leukemia cell subsets was consistent with a lower maturation arrest of AML-*NPM1*^mut^ vs. *NPM1*^wt^ cells, associated with a lower frequency and size of immature leukemia cell expansions (*p* < 0.001), while depicting a higher prevalence of more differentiated AML cells committed to the neutrophil (*p* < 0.001) and/or the monocytic lineage (*p* = 0.02) (Supplementary Table 2 and Supplementary Fig. 1). These findings might contribute to explain the overall higher sensitivity to chemotherapy of AML-*NPM1*^mut^ [7].

Despite AML-*NPM1*^mut^ may originate from CD34^+^ hematopoietic progenitor cells (HPC) [8], most frequently they lack CD34 (7% vs. 94% *NPM1*^wt^ CD34^+^ cells). These cells may expand in BM due to consistent expression of HOX genes [9], which has been directly associated with NPM1 cytoplasmic dislocation [10]. However, CD34^lo^ expression is not specific to AML-*NPM1*^mut^, and it has been found to be independent of NPM1 dislocation [9]. In line with these observations, we also found CD34^lo^ expression among *NPM1*^wt^ immature, neutrophil and monocytic lineage-committed AML cells, and thereby, this phenotype is of limited specificity for AML-*NPM1*^mut^.

Beyond CD34^lo^ expression, other immunophenotypic features of AML-*NPM1*^mut^ cells supported a less pronounced maturation blockade vs. other AML patients. Hence, AML-*NPM1*^mut^ immature cells retained a higher capability for neutrophil lineage maturation with higher expression of CyMPO (*p* = 0.04), CD15 and CD33 (*p* < 0.001), associated with downregulation of the early monocytic markers CD64 (*p* = 0.05) and HLA-DR (*p* = 0.004), in line with the higher frequency of neutrophil (vs. monocytic) lineage-commitment observed for AML-*NPM1*^mut^ cells. Furthermore, AML-*NPM1*^mut^ cases more frequently showed immature AML cells with aberrant CD7 positivity (60% vs. 32% cases), but they rarely expressed CD56 (1% vs. 15%) and NuTdT (3% vs. 20%, respectively) (*p* < 0.001) (Supplementary Fig. 2 and Supplementary Table 3). Multivariate logistic regression analysis revealed that decreased CD34 and HLA-DR, together with upregulation of CD15 and CD7 (but not NuTdT), was the best combination of markers expressed on immature leukemia cells to predict for *NPM1*^mut^ in AML (Table 1).

**Table 1:** Univariate and multivariate logistic regression analysis of those immunophenotypic features of BM leukemia cells associated with *NPM1* mutation (**A**) and *FLT3*-ITD (**B**) from AML patients (*n* = 377).

	AML*-NPM1*^mut^ vs. AML*-NPM1*^wt^ and APL					
(A) Variables and leukemia cell subsets	Univariate analysis			Multivariate analysis		
	OR	95% CI	*p*-value	OR	95% CI	*p*-value
**CD34+ and/or CD117**+**HLADR+ leukemia cells**						
<26.5% of all leukemia cells	2.0	1.4–2.5	<0.001			
CD34 (<35%)	4.8	2.6–8.6	<0.001	7.7	3.5–17.0	<0.001
CD33 (>96%)	1.4	1.0–2.0	0.04			
CD105 (<9.5%)	0.4	0.2–0.6	0.001			
HLA-DR (<97%)	0.3	0.3–0.7	0.001	0.2	0.1–0.5	<0.001
CD15 (>6.6%)	0.3	0.1–0.6	<0.001	0.2	0.1–0.5	<0.001
CD7 (>3%)	1.5	1.0–2.2	0.02	2.0	1.1–3.8	0.02
CD56 negative	0.3	0.1–0.6	0.002			
NuTdT negative	0.2	0.1–0.5	<0.001	0.2	0.1–0.5	0.002
**Neutrophil-committed leukemia cells**						
>21.5% of all leukemia cells	1.6	1.2–2.2	0.008	-	-	-
CD34 (<5%)	4.0	2.4–6.6	<0.001	7.0	2.9–17.5	<0.001
CD71 (<70%)	2.5	1.6–3.9	<0.001	3.0	1.1–7.9	0.02
CD105 (>3%)	5.1	2.7–9.8	<0.001	4.8	1.9–12.4	0.001
CD64 (<30%)	4.3	2.5–7.5	<0.001	4.4	1.8–10.9	0.001
CD13 (<92%)	3.2	2.0–5.1	<0.001	-	-	-
CD56 (>5%)	5.6	2.1–14.5	<0.001	-	-	-
**Monocytic-committed leukemia cells**						
Any asynchronous pattern	6.5	3.8–11.1	<0.001	-	-	-
*Asynchronous CD300e*+*CD14- profile*	85.0	12.0–610	<0.001	49.0	3.7–641	0.004
*Asynchronous CD35*+CD14- profile	11.4	5.5–23	<0.001	-	-	-
CD34+ (<3.8%)	3.7	2.4–5.7	<0.001	-	-	-
Any asynchronous pattern plus CD34+ (<3.8%)	34.3	11.0–108	<0.001	223.9	3.7–641.5	<0.001
CD117 (<5.9%)	3.7	2.1–6.5	0.001	-	-	-
CD13 (<77%)	4.3	2.7–6.8	<0.001	0.2	0.04–1.0	0.05
CD123 (>83%)	2.9	1.8–4.5	<0.001	0.3	0.1–0.8	0.02
CD15+ (>77%)	3.4	2.2–5.4	<0.001	-	-	-
CD36 (>87%)	3.2	2.0–5.1	<0.001	-	-	-
***FLT3-ITD+ vs. FLT3-ITD-***						
**(B) Variables and leukemia cell subsets**						
**AML*-NPM1***^**mut**^ **patients**						
**CD34+ and/or CD117**+**HLADR+ leukemia cells**						
CD34+ (>3%)	5.3	1.9–14.8	0.001	-	-	-
CD38 (<95%)	5.6	2.2–14.1	<0.001	0.1	0.01–0.8	0.03
CD7 (>55%)	5.4	2.2–13.9	<0.001	7.2	1.0–48.5	0.04
CD25 (>25%)	7.1	1.3–37.5	0.02	-	-	-
**Neutrophil-committed leukemia cells**						
CD117 (<69%)	5.7	2.1–15.5	0.001	9.4	2.7–32.4	<0.001
CD123 (>84%)	4.6	1.7–12.6	0.003	7.6	2.2–26.0	0.001
CD13 (>56%)	2.6	1.0–6.8	0.05	-	-	-
**AML*-NPM1***^**wt**^ **patients**						
** % total BM blasts (>40%)**	3.7	1.2–11.6	0.02	-	-	-
**CD34+ and/or CD117**+**HLADR+ leukemia cells**						
CD34+ (<57%)	4.3	1.6–11.5	0.004	3.8	1.0–15.3	0.05
CD25 (>10%)	6.9	1.4–33.8	0.01	7.9	1.5–40.3	0.01

*OR* odds ratio, *CI* confidence interval.

Neutrophil and monocytic lineage-committed AML-*NPM1*^mut^ cells were typically characterized by prominent asynchronous maturation profiles. Thus, despite their CD34^lo^ phenotype, neutrophil lineage AML-*NPM1*^mut^ cells showed (vs. their *NPM1*^wt^ counterpart) more immature features, including downregulation of the neutrophil lineage markers CD15, CD71, CD13 and CD64 (*p* ≤ 0.05), associated with higher levels of the immature antigens CD123 and CD105 (*p* ≤ 0.01). In contrast to *NPM1*^mut^ immature leukemia cells, *NPM1*^mut^ neutrophil-lineage cells barely expressed CD7 but more frequently showed aberrant positivity for CD56 (24% vs. 7%, respectively; *p* = 0.03) and to a lesser extent also for CD9 and CD4 (*p* ≤ 0.02). Furthermore, compared with neutrophil-lineage APL cells, AML-*NPM1*^mut^ neutrophil-lineage cells downregulated CD34, CD13, CD64 and CD71, while they upregulated CD105 (*p* ≤ 0.001), and aberrant CD56 expression, but showed lower rates of CD203c and CD7 (*p* ≤ 0.02) (Supplementary Fig. 2 and Supplementary Table 3). Multivariate analysis revealed that the unique CD34^lo^CD71^lo^CD64^lo^CD105^+^ profile had the highest predictive value for *NPM1*^mut^ among neutrophil lineage AML cells (Table 1).

Finally, *NPM1*^mut^ monocytic-committed leukemia cells showed (vs. AML-*NPM1*^wt^) decreased expression of immature markers (i.e., CD34, CD117; *p* < 0.001), while upregulated the monocytic-associated antigens CD4 (*p* = 0.04), CD11b (*p* = 0.03), CD15, CD36, and CD300e in addition to CD123 (*p* ≤ 0.006). However, these more markedly mature monocytic features coexisted with asynchronous downregulation of other monocytic-associated markers (i.e., CD13, CD71, CD14 and CyMPO; *p* ≤ 0.002) and a higher frequency of AML-*NPM1*^mut^ cases showing aberrant CD56 (*p* = 0.03). Altogether, these phenotypes defined three unique asynchronous monocytic maturation profiles present in most AML-*NPM1*^mut^ cases (90%) vs. a minority of *NPM1*^wt^ patients (24%; *p* < 0.001): [11] (1) abnormal (early) upregulation of CD300e prior to CD14 (CD300e^+^CD14^−^: 74% vs. 3% *NPM1*^wt^ cases, *p* < 0.001); and/or, either (2) early expression of CD35 prior CD14 (CD35^+^CD14^−^: 72% vs. 9%, *p* < 0.001), or; (3) early upregulation of CD14 prior CD35 (CD14^+^CD35^−^: 6% vs. 13%, respectively; *p* = 0.02) (Fig. 1 and Supplementary Table 4). Noteworthy, the presence of CD300e^+^CD14^−^ and/or CD35^+^CD14^−^ leukemia cells showing CD34^lo^ expression emerged as the most specific phenotypes for AML-*NPM1*^mut^ (odds ratio: 223.9; *p* < 0.001) (Table 1).

**Fig. 1:**
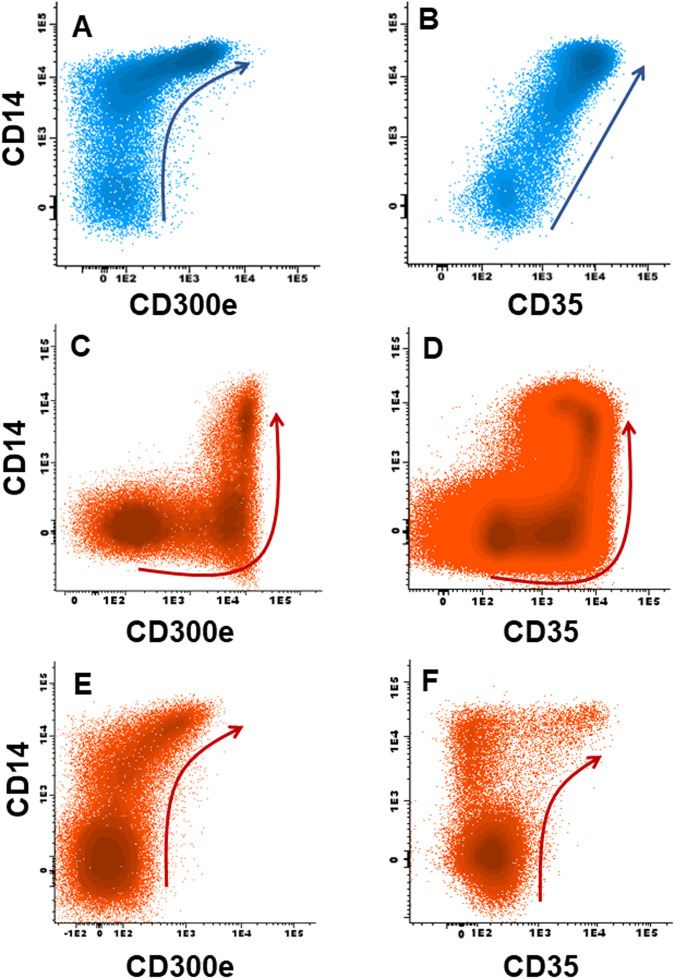
Monocytic maturation pathways in normal and AML bone marrow. Maturation pathways of monocytic cells in normal bone marrow (**A**, **B**, blue dots), and asynchronous AML-*NPM1*^mut^ patterns of expression of CD300e^+^CD14^−^ (**C**), CD35^+^CD14^−^ (**D**). **E** and **F** depict normal patterns of acquisition of CD14 vs. CD300e in a patient with AML-*NPM1*^wt^ while showing an asynchronous CD14^+^ CD35^−^ phenotype (**E**, **F**) among monocytic cells (red dots). Arrows represent the normal (blue) and leukemia (red) maturation pathways of monocytic lineage-committed (gated) CD64^hi^ cells.

Hundreds of neutrophil and monocytic differentiation-associated genes are repressed in AML-*NPM1*^mut^, which might be related to NPM1 haploinsufficiency and/or the cytoplasmic relocation and functional blockade of myeloid transcription factors interacting with NPM1c [12, 13]. For instance, the functional reduction of PU.1 represses the PU.1/CEBPA/RUNX1 myeloid transcriptional hub regulating terminal monocytic and neutrophil differentiation [13]. Conversely, other nuclear transcriptional regulators inhibited by NPM1 under physiological conditions are not translocated to the cytoplasm, leading to abnormally high activation of their target genes [14]. Therefore, asynchronous neutrophil and/or monocytic differentiation profiles of AML-*NPM1*^mut^ cells are consistent with abnormal activation vs. repression of distinct sets of myeloid gene promoters regulated by NPM1.

Expectedly, (non-APL) AML cases with *FLT3*-ITD showed greater BM leukemia cell infiltration and increased proportions of immature CD34^+^ leukemia cells, frequently in association with monocytic AML cells, independently of *NPM1* comutation (Supplementary Fig. 1I and Supplementary Table 2). Such specific expansion of immature AML cells might be related to the physiological restriction of *FLT3* gene expression to BM hematopoietic CD34^+^ HPC [15].

Noteworthy, *FLT3*-ITD promoted distinct immunophenotypic profiles in *NPM1*^mut^ and *NPM1*^wt^ AML (Supplementary results and Supplementary Fig. 3). Although CD34 and/or CD25 expression has been associated with *FLT3*-ITD [6], we show that both markers are more frequent among immature *NPM1*^mut^*FLT3*-ITD^+^ cells. Hence, CD25 positivity and heterogeneous CD34 expression on immature AML cells emerged as the best combination of predictors for *FLT3*-ITD among AML-*NPM1*^wt^ cases. Conversely, in AML-*NPM1*^mut^ cases, *FLT3*-ITD was strongly associated with a CD7^hi^CD38^lo^ profile on immature leukemia cells and/or a CD117^het^CD123^hi^ phenotype among neutrophil lineage leukemia cells (Table 1).

In summary, the mutational status of *NPM1* and *FLT3* is associated with unique BM leukemia cell distribution and immunophenotypic profiles, even when only cases with a normal karyotype were considered (data not shown), which might contribute to a fast diagnostic screening of *NPM1*^mut^ and/or *FLT3*-ITD in AML, and an improved classification of AML-*NPM1*^mut^ patients. Further prospective studies are needed to confirm these findings.

### Supplementary information

Supplementary data

## Data Availability

The datasets generated during and/or analyzed during the current study are available from the corresponding author upon reasonable request.
