# Supplementary material for: Baseline immunophenotypic profile of bone marrow leukemia cells in acute myeloid leukemia with nucleophosmin-1 gene mutation: a EuroFlow study
Source: Blood Cancer J. 2023 Sep 4;13(1):132. doi: 10.1038/s41408-023-00909-4 (PMC10477264; doi:10.1038/s41408-023-00909-4)
Supplement: Supplementary file 1 — Supplementary data [file 41408_2023_909_MOESM1_ESM.pdf]

Supplementary Data.

Baseline immunophenotypic profile of bone marrow leukemia cells in acute myeloid leukemia with nucleophosmin-1 gene mutation: a Euroflow study.

Sergio Matarraz[1,2*], Pilar Leoz[2,3], Ana Yeguas-Bermejo[2,3], Vincent van der Velden[4], Anne E. Bras[4], Jose I. Sánchez Gallego JI[1,2], Quentin Lecrevisse[1,2], Rosa Ayala-Bueno[1,2], Cristina Teodosio[1,2], Ignacio Criado[1,2], María González-González[1,2], Juan Flores-Montero[2,3], Alejandro Avendaño[2,3], María B. Vidriales[2,3], María C. Chillón[2,3], Teresa González[2,3], Ramón García-Sanz[2,3], María I. Prieto Conde[2,3], Neus Villamor[5], Laura Magnano[5], Enrique Colado[6], Paula Fernández[7], Edwin Sonneveld[8], Jan Philippé[9], Michaela Reiterová[10], Juan C. Caballero Berrocal[11], Francisco J. Diaz-Gálvez[12], Fernando Ramos[13], Julio Dávila Valls[14], Raquel Manjón Sánchez[15], Jackeline Solano Tovar[16], Xavier Calvo[17], Luis García Alonso[18], Leonor Arenillas[17], Sara Alonso[6], Ariana Fonseca[6], Covadonga Quirós Caso[6], Jacques J.M. van Dongen[1,2,19], Alberto Orfao[1,2*].

Running title. Immunophenotypic profile of NPM1 mutated AML

Author Affiliations:

[1]Translational and Clinical Research Program, Centro de Investigación del Cáncer (IBMCC; CSIC-University of Salamanca); Cytometry Service, NUCLEUS; Department of Medicine, University of Salamanca (USAL) and Institute of Biomedical Research of Salamanca (IBSAL).

[2] Biomedical Research Networking Centre Consortium of Oncology (CIBERONC), Instituto de Salud Carlos III, 28029 Madrid, Spain.

[3]Hematology Department, University Hospital of Salamanca, CIBERONC (CB16/12/00233), IBSAL, Accelerator program and Centro de Investigación del Cáncer (IBMCC; CSIC-University of Salamanca).

[4]Department of Immunology, Erasmus MC, University Medical Center Rotterdam, Rotterdam, The Netherlands.

[5]Hematology Service, Hospital Clinic, Barcelona, Spain.

[6]Hematology Department and Laboratory Medicine Department, Hospital Universitario Central de Asturias, Oviedo, Spain.

[7]FACS/Stem Cell Laboratory, Kantonsspital Aarau, Aarau, Switzerland.

[8]Dutch Childhood Oncology Group, The Hague, The Netherlands.

[9]Department of Diagnostic Sciences, Faculty of Medicine and Health Sciences, Ghent University, Ghent, Belgium.

[10]CLIP-Department of Pediatric Hematology and Oncology, Second Medical Faculty, Charles University and University Hospital Motol, Prague, Czech Republic.

[11]Department of Hematology, Hospital Clínico Universitario de Valladolid, Valladolid, Spain; [12]Department of Hematology, Hospital Universitario de Burgos, Burgos, Spain.

[13]Department of Hematology, Complejo Asistencial Universitario de León, León, Spain.
[14]Department of Hematology, Complejo Asistencial de Ávila, Ávila, Spain.
[15]Department of Hematology, Complejo Asistencial de Zamora, Zamora, Spain.
[16]Department of Hematology, Complejo Asistencial Universitario de Palencia, Palencia, Spain.
[17]Pathology Service, Hospital del Mar, Barcelona, Spain.
[18]Hematology Service, University Hospital of Getafe, Madrid, Spain;
[19]Department of Immunology, Leiden University Medical Center, Leiden, The Netherlands.

Supplementary Methods.

Patients and samples. A total of 377 BM samples from newly-diagnosed, previously untreated (unless stated otherwise), adult (n=366, 55% males and 45% females; median age of 62 years, range: 19-90 years) and pediatric AML patients (n=11, 36% males and 64% females; median age 12 years, range: 1-15 years) diagnosed according to the World Health Organization (WHO) 2016 criteria, were retrospectively studied.[1] These included 201 AML cases with *NPM1*^{mut} together with 144 AML-*NPM1*^{wt} and 32 patients with APL. *FLT3*-ITD was systematically investigated and it was detected in 66/201 AML-*NPM1*^{mut} cases (33%), 27/144 (19%) AML-*NPM1*^{wt} and in 11/32 APL (34%) patients.

According to the WHO classification,[1] AML-*NPM1*^{wt} (non-APL) patients were distributed as follows: i) 42 had AML with recurrent genetic abnormalities -4 AML with t(8;21)(q22;q22.1) cases, 7 AML with inv(16)(p13.1q22) or t(16;16)(p13.1;q22), 6 AML with t(9;11)(p21.3;q34.1), 1 AML with t(11;19)(q23.3;p13.3), 1 AML with t(6;9)(p23;q34.1), 4 AML with inv(3)(q21.3q26.2) or t(3;3)(q21.3;q26.2), 4 AML with biallelic *CEBPA* gene mutation, and 15 AML with mutated *RUNX1*-; ii) 42 AML with myelodysplasia-related changes; iii) 9 therapy-related myeloid neoplasms; iv) 51 AML-NOS (not otherwise specified) (6 AML with minimal differentiation, 17 AML without maturation, 13 AML with maturation, 2 acute myelomonocytic leukemias; 12 acute monoblastic and monocytic leukemias and 1 pure erythroid leukemia). All patients gave their informed consent to participate in the study according to the Declaration of Helsinki, and the study was approved by the Ethics committees of the 16 participant centers (Salamanca, Barcelona, Asturias, Valladolid, Burgos, León, Ávila, Zamora, Palencia and Getafe, Spain; Rotterdam, The Hague and Leiden, The Netherlands; Aarau, Switzerland; Ghent, Belgium and; Prague, Czech Republic).

Immunophenotypic studies. Flow cytometry immunophenotyping was performed at diagnosis at the center of origin on freshly-obtained (<36 h) EDTA or heparin anticoagulated BM samples, using EuroFlow standard operating procedures.[2,3] Thus, BM samples were stained with the 8-color EuroFlow acute leukemia orientation tube (ALOT) and the EuroFlow AML/MDS antibody panel (Supplementary Table 1), as described in detail at www.euroflow.org.[4] Stained cells were measured locally in FACSCanto II flow cytometers (Becton/Dickinson Biosciences, BD; San Jose, CA) equipped with the FACSDiva 6.1 software (BD). Subsequently, flow cytometry data files were analysed both locally and centrally at the Cytometry Service of the University of Salamanca (Salamanca, Spain) using Infinicyt (software version 2.0.5; Cytognos SL, Salamanca, Spain). For inclusion in the study, each patient data file underwent stringent quality assessment criteria, as previously described.[5] In each case, leukemia cells were identified using their unique immunophenotypic profile for the four backbone markers (CD34, HLA-DR, CD117 and CD45) and light scatter characteristics on bivariate dot plots. Analysis of leukemia cells in the ALOT tube, was performed as previously reported.[6] Presence of an abnormal expansion of a BM cell compartment was established when its mean frequency was >2 SD from its frequency in BM from healthy subjects.[7]

Interphase fluorescence in situ hybridization studies. Interphase fluorescence in situ hybridization aimed at detection of chromosomal rearrangements and translocations, was performed on interphase nuclei from whole BM cells after they had been fixed in 3/1 (v/v) methanol/acetic, according to previously reported techniques.[8] For this purpose, the following chromosomal probes purchased from Vysis (Downers Grove, IL) and Kreatech, (Amsterdam, The Netherlands) were used in double stainings (spectrum orange and spectrum green): i) LSI RUNX1/RUNX1T1 Dual Color (DC) Dual Fusion (DF) (Vysis) for t(8;21)(q22;q22.1); ii) LSI CBFB DC Break Apart (BA) (Vysis) for inv(16)(p13.1q22) or t(16;16)(p13.1;q22); iii) LSI PML/RARA DC, DF (Vysis) for t(15;17)(q24;q21.1) and LSI RARA DC BA (Vysis) for rearrangements of the RARA gene; iv) LSI MLL DC BA (Vysis) for t(9;11)(p21.3;q34.1) and t(11;19)(q23.3;p13.3); v) DEK/NUP214 t(6;9) DC DF (Kreatech) for t(6;9)(p23;q34.1); vi) EVI t(3;3); inv(3) (3q26) DC BA (Kreatech) for inv(3)(q21.3q26.2) or t(3;3)(q21.3;q26.2).

Genetic and molecular studies. Interphase fluorescence in situ hybridization aimed at detection of chromosomal rearrangements and translocations was performed according to previously reported techniques.[8] In parallel, presence of *RUNX1::RUNX1T1*, *CBFB::MYH11* and *PML::RARA* fusion transcripts was assessed by real-time quantitative polymerase chain reaction (RQ-PCR) according to the recommendations of the Europe Against Cancer Program.[9] Mutations involving *FLT3*-ITD, *NPM1* and *CEBPA* were determined by fragment analysis and/or Sanger sequencing, following previously reported probes and protocols.[10–12] In addition to the above genetic analyses, next generation sequencing (NGS) based on a custom captured-based gene panel (PanMyeloid Panel, SOPHiA GENETICS, Switzerland) was performed.[13] Sequences obtained were analysed with the SOPHIA GENETICS DDM v3.0 software (Lausanne, Switzerland).

Statistical methods. Median (range) and mean (SD) values, as well as the 10th, 25th, 75th and 90th percentiles, were calculated for continuous variables; for categorical variables, frequencies were reported. For all immunophenotypic markers investigated, a cut-off for positivity of ≥20% expression on specific cell populations was used. To determine the statistical significance of differences observed among ≥2 groups, the Mann–Whitney U and the Kruskal–Wallis tests (for continuous variables) or the X^2-test (for categorical variables), were used. Receiver operating curves were employed to establish cut-off values to predict for *NPM1* mutation. Odds ratios (OR) and their 95% confidence intervals (CI) were calculated for the informative immunophenotypic parameters using univariate and multivariate logistic regression models. *P*-values <0.05 (with a false discovery rate correction for multiple comparisons of <5%) were considered statistically significant. For statistical analyses, the SPSS 26.0 software (SPSS, IBM, Chicago, IL) was used. The GraphPad Prism 8 (version 8.0.2; GraphPad Software Inc., Boston, MA) was used for box-plot figures.

Supplementary Results.

Immunophenotype of AML-*NPM1*^{mut} leukemia cell subsets in BM. Among *NPM1*^{mut} patients, immature AML cells showed higher expression of CD34 (median of 7% vs. 0% AML cells), CD117 (100% vs. 87%), HLA-DR (90% vs. 0%), CD71 (77% vs. 44%), CD13 (96% vs. 62%), CD123 (96% vs. 83%), CD38 (95% vs. 84%) and CD15 (23% vs. 9%) compared to AML-*NPM1*^{mut} neutrophil lineage-committed leukemia cells ($p<0.001$), together with lower levels of CD105 (2% vs. 10%, $p<0.001$), CyMPO (60% vs. 88%, $p<0.001$) and CD64 (7% vs. 13%; $p=0.03$) (Figure 2). In addition, compared to *NPM1*^{mut} neutrophil lineage-committed leukemia cells, AML-*NPM1*^{mut} immature cells more frequently showed CD4 (16% vs. 6%, $p<0.001$) and aberrant CD7 (38% vs. 0%, $p<0.001$), CD25 (6% vs. 0.03%, $p<0.001$) and CD9 expression (23% vs. 8% AML cells; $p=0.02$) in the absence of CD56 expression (1% vs. 24% AML cases, respectively; $p<0.001$) (Supplementary Figure 2, Supplementary Table 3).

In contrast to immature and neutrophil lineage-committed AML-*NPM1*^{mut} cells, AML-*NPM1*^{mut} monocytic leukemia cells systematically expressed (>90% cells) CD64^{hi}, HLA-DR^{+}, CD33^{hi}, CD38^{+}, CD36^{+}, CD15^{+} and CD123^{lo}, together with CD11b^{+} (81%), CD4^{lo} (80%), CD35^{+} (48%), CD300e^{+} (45%) and CD14 (31%) in a significant fraction of these cells (Supplementary Figure 2). In turn, they showed lower expression of CD34 (median of 0% vs. 7% and 0% AML cells), CD117 (5% vs. 100% and 87%), CD71 (34% vs. 77% and 44%), CD13 (37% vs. 96% and 62%), CyMPO (34% vs. 60% and 88%), associated with a higher frequency of CD105^{+} (39% vs. 2% and 10%; $p<0.001$) and CD9^{+} leukemia cells (37% vs. 23% and 8%, respectively; $p<0.001$) (Supplementary Figure 2). In addition, aberrant CD56 expression was more frequently detected among AML-*NPM1*^{mut} patients with monocytic-lineage leukemia cells vs. those with immature and neutrophil lineage-committed AML cells (46% vs. 1% and 24% of AML cases, respectively; $p<0.001$), while expression of CD7, CD22, NG2, CD25 and NuTdT on monocytic leukemia cells was infrequent (≤2% of AML cases) (Supplementary Figure 2 and Supplementary Table 3).

Phenotypic profiles of AML-*NPM1*^{mut} *FLT3*-ITD^{+} vs. *FLT3*-ITD^{-} cells. Coexistence of *NPM1*^{mut} and *FLT3*-ITD^{+} in AML was associated with an enhanced BM leukemia cell infiltration vs. AML *NPM1*^{mut}*FLT3*-ITD^{-} cases (87% vs. 68% AML cells; $p<0.001$), and a greater expansion of

immature AML cells (61% vs. 39% cases, respectively; p=0.008) (Supplementary Figure 1-I), which accounted for a median percentage of 36% vs. 25% of all leukemia cells, respectively (p=0.05) (Supplementary Table 2). Such expansion of immature AML cells more frequently occurred in patients who also showed monocytic AML cells (35% vs. 17% cases; p<0.001), while decreased the frequency of AML *NPM1*^{mut}*FLT3*-ITD^{+} cases who had a predominant monocytic leukemia cell expansion (10% vs. 23%; p=0.04) and mixed expansions of neutrophil and monocytic AML cells (1% vs. 10% cases, respectively; p=0.03) (Supplementary Figure 1-I).

The coexistence of *NPM1*^{mut} and *FLT3*-ITD^{+} on immature BM leukemia cells was associated with unique immunophenotypes including: upregulation (vs. *NPM1*^{mut}*FLT3*-ITD^{-} cells) of stem cell-associated and aberrant markers, like CD34 (10% vs. 3% positive cells, p=0.04), CD123 (98% vs. 94%, p=0.01), CD7 (64% vs. 18%, p<0.001), CD25 (28% vs. 0%, p=0.01) and CD22 (18% vs. 4% positive cases, p=0.02), together with lower CD38 levels (90% vs. 97% positive cells, respectively p=0.001) (Supplementary Figure 3). Similarly, *NPM1*^{mut}*FLT3*-ITD^{+} neutrophil lineage-committed AML cells also showed more immature and aberrant phenotypes vs. their *NPM1*^{mut}*FLT3*-ITD^{-} counterpart, including: upregulation of CD123 (94% vs. 76%, p=0.001), CD105 (18% vs. 4%, p=0.002), CD13 (85% vs. 43%, p=0.002), and an increased frequency of patients expressing CD56 (35% vs. 20%, p=0.02), CD22 (8% vs. 2%, p=0.03) and CD25 (4% vs. 0%, respectively; p=0.05) (Supplementary Figure 3). However, these *NPM1*^{mut}*FLT3*-ITD^{+} cells depicted (vs. *FLT3*-ITD^{-} cases) asynchronous CD117 downregulation (65% vs. 92% positive AML cells, p=0.002) and a slightly higher CD11b reactivity (8% vs. 3%, respectively; p=0.02). However, no significant phenotypic differences were observed among monocytic cells from *NPM1*^{mut} *FLT3*-ITD^{+} vs. *NPM1*^{mut}*FLT3*-ITD^{-} AML cases (Supplementary Table 4 and Supplementary Figure 3).

Phenotypic profiles of AML-*NPM1*^{wt} and APL patients with *FLT3*-ITD^{+}. Interestingly, also among *NPM1*^{wt} AML cases, the presence of *FLT3*-ITD^{+} was associated with a higher percentage of BM infiltration by leukemia cells (74% vs. 51% among *NPM1*^{wt}*FLT3*-ITD^{-} AML cases, p=0.008) (Supplementary Table 2). Of note, this overall AML cell increase also affected their relative distribution and phenotypic profiles, as reflected by a higher frequency of *NPM1*^{wt}*FLT3*-ITD^{+} cases with mixed expansions of immature plus neutrophil lineage-committed AML cells (19% vs. 8% *NPM1*^{wt}*FLT3*-ITD^{-} cases; p=0.001), and of AML *NPM1*^{wt}*FLT3*-ITD^{+} cases with a predominant

monocytic population (22% vs. 8% *NPM1*^{wt}*FLT3*-ITD^{-} cases; p=0.001) (Supplementary Figure 1). Compared to *NPM1*^{wt}*FLT3*-ITD^{-} AML, *NPM1*^{wt}*FLT3*-ITD^{+} cases also showed lower CD34 expression on immature leukemia cells (49% vs. 97% cells, p=0.01), downregulation of CD71 both on neutrophil (39% vs. 76%, p=0.05) and monocytic lineage-committed leukemia cells (16% vs. 47%, p=0.05), and increased positivity for CD25 on both immature (30% vs. 7%, p=0.009) and monocytic AML cells (8% vs. 0%, p=0.005) (Supplementary Figure 2).

Similarly, APL cases presenting with *FLT3*-ITD^{+} showed a significantly higher percentage of cases with a predominant immature leukemia cell compartment (18% vs. 0% APL *FLT3*-ITD^{-} cases; p=0.04), associated with a higher median percentage of immature leukemia cells (3% vs. 0.3% cells; p=0.03) vs. *FLT3*-ITD^{-} cases; despite this, these two groups of APL patients showed an overall similar level of BM infiltration by leukemia cells (80% vs. 76% APL *FLT3*-ITD^{-} cases, respectively; p>0.05) (Supplementary Table 2 and Supplementary Figure 1).

Univariate analysis of *NPM1* mutation-associated immunophenotypes in AML. Univariate logistic regression analysis revealed that AML-*NPM1*^{mut} was associated with the presence of a lower percentage (<26.5%) of immature leukemia cells -odds ratio (OR): 2.0, p<0.001- showing also lower expression of CD34 (<35% positive cells; OR, 4.8; p<0.001), CD105 (<9.5%; OR, 0.4; p=0.001) and HLA-DR (<97%; OR, 0.3; p=0.001), together with expression of CD15 (>6.6%; OR, 0.3; p<0.001), CD33 (>96%; OR, 1.4; p=0.04), and aberrant positivity for CD7 (OR, 1.5; p=0.02) in the absence of NuTdT (OR, 0.2, p<0.001) and CD56 expression (OR, 0.3, p=0.002) (Table 1). In addition, AML-*NPM1*^{mut} was further characterized by higher numbers of neutrophil lineage-committed leukemia cells (>21.5%; OR, 1.6; p=0.008) displaying low levels of CD34 (<5% positive cells; OR, 4.0; p<0.001), CD71 (<70%; OR, 2.5; p<0.001), CD64 (<30%; OR, 4.3; p<0.001) and CD13 (<92%; OR, 3.2, p<0.001) and positivity for both CD105 (>3%; OR, 5.1; p<0.001) and CD56 (>5%; OR, 5.6; p<0.001). Regarding monocytic-lineage AML cells, the highest predictive value for *NPM1*^{mut} in AML was associated with the presence of any asynchronous pattern in monocytic AML cells (OR, 6.5; p<0.001), including the CD300e^{+} CD14^{-} (OR, 85.0; p<0.001) and CD35^{+} CD14^{-} (OR, 11.4; p<0.001) expression profiles, low CD34 (OR, 3.8; p<0.001), simultaneous presence of any asynchronous monocytic pattern and low CD34 (OR, 34.3; p<0.001), decreased CD13 (<77% positive AML cells; OR, 4.3; p<0.001) and CD117 expression (<5.9%; OR, 3.7;

p=0.001) and high reactivity for CD15 (>77%; OR, 3.4; p<0.001), CD36 (>87%; OR, 3.2; p<0.001), and CD123 (>83%; OR, 2.9; p<0.001) (Table 1).

When we restricted the analysis to cases showing a normal karyotype, similar phenotypic differences were observed (data not shown).

Univariate analysis of *FLT3*-ITD associated immunophenotypes in *NPM1*^{mut} and *NPM1*^{wt} AML. Univariate analysis revealed that the presence of *FLT3*-ITD on AML-*NPM1*^{mut} patients was associated with the presence of immature AML cells showing positivity for CD34 (OR, 5.3; p=0.001), CD7 (>55% positive AML cells; OR, 5.4; p<0.001) and CD25 (>25%; OR, 7.1; p=0.02), together with heterogeneous CD38 expression levels (<95%; OR, 5.6; p<0.001) (Table 1). Among AML-*NPM1*^{mut} patients with neutrophil lineage leukemia cells, *FLT3*-ITD was associated with heterogeneous CD117 expression (<69% positive AML cells; OR, 5.7; p=0.001) and high levels of both CD123 (>84%; OR, 4.6; p=0.003) and CD13 expression (>56%; OR, 2.6; p=0.05). In contrast, no phenotypic features of monocytic-committed AML-*NPM1*^{mut} cells were found to be associated with coexistence of *FLT3*-ITD (Table 1).

Among AML-*NPM1*^{wt} patients baseline detection of higher BM leukemia cell counts (>40%; OR, 3.7, p=0.02) with *NPM1*^{wt} immature blasts showing lower and heterogeneous CD34 expression (<57%, OR, 4.3; p=0.004) together with higher positivity for CD25 (>10%; OR, 6.9; p=0.01) was associated with *FLT3*-ITD, in the univariate analysis (Table 1).

Supplementary references

1 Arber DA, Orazi A, Hasserjian R, Thiele J, Borowitz MJ, Le Beau MM *et al.* The 2016 revision to the World Health Organization classification of myeloid neoplasms and acute leukemia. *Blood* 2016; **127**: 2391–2405.

2 Kalina T, Flores-Montero J, Lecrevisse Q, Pedreira CE, van der Velden VHJ, Novakova M *et al.* Quality assessment program for EuroFlow protocols: summary results of four-year (2010-2013) quality assurance rounds. *Cytom Part J Int Soc Anal Cytol* 2015; **87**: 145–156.

3 Kalina T, Flores-Montero J, van der Velden VHJ, Martin-Ayuso M, Böttcher S, Ritgen M *et al.* EuroFlow standardization of flow cytometer instrument settings and immunophenotyping protocols. *Leukemia* 2012; **26**: 1986–2010.

4 van Dongen JJM, Lhermitte L, Böttcher S, Almeida J, van der Velden VHJ, Flores-Montero J *et al.* EuroFlow antibody panels for standardized n-dimensional flow cytometric immunophenotyping of normal, reactive and malignant leukocytes. *Leukemia* 2012; **26**: 1908–1975.

5 Bras AE, Matarraz S, Nierkens S, Fernández P, Philippé J, Aanei C-M *et al.* Quality Assessment of a Large Multi-Center Flow Cytometric Dataset of Acute Myeloid Leukemia Patients-A EuroFlow Study. *Cancers* 2022; **14**: 2011.

6 Lhermitte L, Mejstrikova E, van der Sluijs-Gelling AJ, Grigore GE, Sedek L, Bras AE *et al.* Automated database-guided expert-supervised orientation for immunophenotypic diagnosis and classification of acute leukemia. *Leukemia* 2018; **32**: 874–881.

7 Orfao A, Matarraz S, Pérez-Andrés M, Almeida J, Teodosio C, Berkowska MA *et al.* Immunophenotypic dissection of normal hematopoiesis. *J Immunol Methods* 2019; **475**: 112684.

8 Orfao A, Chillón MC, Bortoluci AM, López-Berges MC, García-Sanz R, Gonzalez M *et al.* The flow cytometric pattern of CD34, CD15 and CD13 expression in acute myeloblastic leukemia is highly characteristic of the presence of PML-RARalpha gene rearrangements. *Haematologica* 1999; **84**: 405–412.

9 Gabert J, Beillard E, van der Velden VHJ, Bi W, Grimwade D, Pallisgaard N *et al.* Standardization and quality control studies of 'real-time' quantitative reverse transcriptase polymerase chain reaction of fusion gene transcripts for residual disease detection in leukemia - a Europe Against Cancer program. *Leukemia* 2003; **17**: 2318–2357.

10 Thiede C, Koch S, Creutzig E, Steudel C, Illmer T, Schaich M *et al.* Prevalence and prognostic impact of NPM1 mutations in 1485 adult patients with acute myeloid leukemia (AML). *Blood* 2006; **107**: 4011–4020.

11 Thiede C, Steudel C, Mohr B, Schaich M, Schäkel U, Platzbecker U *et al.* Analysis of FLT3-activating mutations in 979 patients with acute myelogenous leukemia: association with FAB subtypes and identification of subgroups with poor prognosis. *Blood* 2002; **99**: 4326–4335.

12 Benthaus T, Schneider F, Mellert G, Zellmeier E, Schneider S, Kakadia PM *et al.* Rapid and sensitive screening for CEBPA mutations in acute myeloid leukaemia. *Br J Haematol* 2008; **143**: 230–239.

13 Aguilera-Diaz A, Vazquez I, Ariceta B, Mañú A, Blasco-Iturri Z, Palomino-Echeverría S *et al.* Assessment of the clinical utility of four NGS panels in myeloid malignancies. Suggestions for NGS panel choice or design. *PloS One* 2020; **15**: e0227986.

Supplementary Table 1. Euroflow AML/MDS panel: antibody positions per fluorochrome

Tube	Fluorochrome conjugates							
	**PacB**	**PacO**	**FITC**	**PE**	**PerCPCy5.5**	**PECy7**	**APC**	**APCH7**
**1**	HLADR	CD45	CD16	CD13	CD34	CD117	CD11b	CD10
**2**	HLADR	CD45	CD35	CD64	CD34	CD117	CD300e	CD14
**3**	HLADR	CD45	CD36	CD105	CD34	CD117	CD33	CD71
**4**	HLADR	CD45	NuTdT	CD56	CD34	CD117	CD7	CD19
**5**	HLADR	CD45	CD15	NG2	CD34	CD117	CD22	CD38
**6**	HLADR	CD45	CD42a and CD61	CD203c	CD34	CD117	CD123	CD4
**7**	HLADR	CD45	CD41	CD25	CD34	CD117	CD42b	CD9

AML, acute myeloid leukemia; APC, allophycocyanine; Cy7, cyanine7; FITC, fluorescein isothiocyanate; H7, hilite7; MDS, myelodysplastic syndrome; Nu, nuclear; PacB, pacific blue; PacO, pacific orange; PE, phycoerythrin; PerCPCy5.5, peridinin–chlorophyll–protein–cyanine 5.5.

Supplementary Table 2. Distribution of different subsets of leukemia cells in BM of AML and APL patients according to the presence vs. absence of *NPM1*^{mut} and/or *FLT3-ITD*

**Phenotypic subsets of leukemia cells**	**AML patient groups**											
	**AML-*NPM1*^{wt}**				**AML-*NPM1*^{mut}**				**APL**			
	***FLT3-ITD*$^{-}$ (n=117)**	***FLT3-ITD*$^{+}$ (n=27)**	**Total *NPM1*^{wt} (n=144)**	**p $^{\#}$**	***FLT3-ITD*$^{-}$ (n=135)**	***FLT3-ITD*$^{+}$ (n=66)**	**Total *NPM1*^{mut} (n=201)**	**P $^{\#}$**	***FLT3-ITD*$^{-}$ (n=21)**	***FLT3-ITD*$^{+}$ (n=11)**	**Total APL (n=32)**	**P $^{\#}$**
% total BM leukemia cells	51% (9-97%)**	74% (13-97%)**	55% (9-97%)**	**0.008**	68% (8-8%)	87% (19-99%)	75% (8-98%)	**<0.001**	76% (26-98%)	80% (71-87%)	79% (26-98%)	**0.4**
**Immature leukemia cells**	63% (0-100%)**	56% (0-100%)**	62% (0-100%)**	**0.4**	25% (0-100%)	36% (0-100%)	26% (0-100%)	**0.05**	.3% (.05-15)*	3% (0-26%)	.5% (0-26%)**	**.03**
**Neutrophil-lineage leukemia cells**	11% (0-100)**	12% (0-80%)**	11% (0-100)**	**0.9**	37% (0-100%)	34% (0-100%)	36% (0-100%)	**0.8**	99% (85-100%)**	88% (70-100%)**	99% (70-100%)**	**.003**
**Monocytic leukemia cells**	26% (0-100)*	32% (0-100)	27% (0-100%)	**0.9**	38% (0-100%)	30% (0-100%)	35% (0-100%)	**0.08**	.2% (0-2)**	2% (.05-13)	.5% (0-13)**	**.05**

Results expressed as median percentage (range) of leukemia cells in BM. #, p-values correspond to comparisons between *FLT3*-ITD- and *FLT3*-ITD+ AML patient groups; *, $p<0.05$; **, $p<0.03$ vs. *NPM1*^{mut} cases. AML, acute myeloid leukemia; APL, acute promyelocytic leukemia; BM, bone marrow; ns, no statistically significant differences detected.

Supplementary Table 3. Immunophenotypic patterns of leukemia cell subsets in AML patients

Leukemic cell phenotype	CD34+ and/or CD117+HLADR+ AML cells (n=212)			AML cells with Neutrophil maturation (n=150)				AML cells with monocytic maturation (n=165)		
	$NPM1^{wt}$ (n=120)	$NPM1^{mut}$ (n=92)	*p-value**	$NPM1^{wt}$ (n=28)	$NPM1^{mut}$ (n=90)	*APL (n=32)*	*p-value**	$NPM1^{wt}$ (n=58)	$NPM1^{mut}$ (n=107)	*p-value**
**Hematopoietic precursor cell markers**										
CD34	105 (88%)	23 (25%)	***<0.001***	12 (43%)	5 (6%)	15 (47%)	***<.001***	20 (35%)	1 (1%)	***<.001***
CD33	113 (94%)	92 (100%)	***0.02***	26 (93%)	87 (97%)	31 (97%)	***ns***	58 (100%)	107 (100%)	***ns***
CD38	117 (98%)	92 (100%)	***ns***	24 (86%)	89 (99%)	27 (84%)	***0.004***	57 (98%)	107 (100%)	***ns***
CD71	120 (100%)	92 (100%)	***ns***	25 (89%)	80 (89%)	27 (84%)	***ns***	40 (69%)	72 (67%)	***ns***
CD105	32 (27%)	5 (5%)	***<0.001***	5 (18%)	26 (29%)	0%	***0.002***	36 (62%)	71 (66%)	***ns***
CD117	120 (100%)	92 (100%)	***ns***	26 (93%)	89 (99%)	31 (97%)	***ns***	18 (31%)	10 (9%)	***<.001***
CD123	119 (99%)	91 (99%)	***ns***	22 (79%)	83 (92%)	29 (91%)	***ns***	58 (100%)	107 (100%)	***ns***
HLADR	120 (100%)	92 (100%)	***ns***	0%	0%	0%	***ns***	55 (95%)	105 (98%)	***ns***
**Myeloid associated markers**										
CyMPO	78 (65%)	76 (83%)	***.01***	27 (96%)	70 (78%)	31 (97%)	**0.01**	47 (82%)	70 (66%)	***0.03***
CD11b	0%	0%	***ns***	4 (14%)	4 (4%)	2 (6%)	***ns***	52 (90%)	97 (91%)	***ns***
CD13	119 (99%)	88 (96%)	***ns***	26 (93%)	77 (86%)	31 (97%)	***ns***	52 (90%)	68 (64%)	***<.001***
CD15	46 (38%)	52 (56%)	***0.008***	15 (54%)	29 (32%)	15 (47%)	***ns***	57 (98%)	107 (100%)	***ns***
CD16	0%	0%	***ns***	0%	0%	0%	***ns***	1 (2%)	11 (10%)	***.05***
CD14	0%	0%	***ns***	0%	0%	0%	***ns***	46 (79%)	69 (64%)	***0.04***
CD35	0%	0%	***ns***	2 (7%)	2 (2%)	1 (3%)	***ns***	45 (78%)	89 (83%)	***ns***
CD36	13 (11%)	4 (4%)	***ns***	3 (11%)	0%	0%	***.001***	53 (91%)	105 (98%)	***0.05***
CD64	49 (41%)	25 (27%)	***.03***	15 (54%)	34 (38%)	30 (94%)	***<.001***	58 (100%)	107 (100%)	***ns***
CD300e	0%	0%	***ns***	0%	0%	0%	***ns***	34 (59%)	89 (83%)	***0.001***
**Lymphoid and aberrant markers**										
CD4	50 (42%)	38 (41%)	***ns***	5 (18%)	22 (24%)	7 (22%)	***ns***	51 (88%)	102 (95%)	***ns***
CD7	38 (32%)	55 (60%)	***<0.001***	6 (21%)	5 (6%)	6 (19%)	***.02***	1 (2%)	2 (2%)	***ns***
CD19	11 (9%)	7 (8%)	***ns***	0%	1 (1%)	0%	***ns***	0%	0%	***ns***
CD22	14 (12%)	9 (10%)	***ns***	1 (4%)	3 (3%)	0%	***ns***	1 (2%)	2 (2%)	***ns***
CD203c	0%	0%	***ns***	0%	0%	7 (22%)	***<.001***	0%	0%	***ns***
CD56	18 (15%)	1 (1%)	***<0.001***	2 (7%)	22 (24%)	3 (9%)	***0.04***	17 (29%)	49 (46%)	***0.03***
nuTdT	24 (20%)	3 (3%)	***<0.001***	1 (4%)	6 (7%)	0%	***ns***	1 (2%)	1 (1%)	***ns***
NG2	0%	0%	***ns***	0%	0%	0%	***ns***	5 (9%)	2 (2%)	***.05***
**Other evaluated markers**										
CD25	33/105 (32%)	12/33 (36%)	***ns***	1/24 (4%)	1/39 (3%)	0/7 (0%)	***ns***	3/58 (5%)	1/44 (2%)	***ns***
CD9	59/105 (56%)	18/33 (54%)	***ns***	5/24 (21%)	9/39 (23%)	4/7 (57%)	***ns***	32/58 (55%)	28/44 (64%)	***ns***

Results expressed as number of AML cases (percentage between brackets). *, for comparisons among *groups*. AML, acute myeloid leukemia; ns, no statistically significant differences detected.

Supplementary Table 4. Frequency of asynchronous immunophenotypic maturation profiles detected among monocytic leukemia cells in BM of AML patients.

	**AML-*NPM1*^{wt}**			**AML-*NPM1*^{mut}**			
	FLT3-ITD^{-} **(n=117)**	***FLT3-ITD***$^{+}$ **(n=27)**	**Total *NPM1*^{wt} (n=144)**	***FLT3-ITD***$^{-}$ **(n=135)**	***FLT3-ITD***$^{+}$ **(n=66)**	**Total *NPM1*^{mut} (n=201)**	****p-value***
**Asynchronous monocytic patterns**	29 (25%)	6 (22%)	35 (24%)	120 (89%)	60 (91%)	180 (90%)	<0.001
Asynchronous (early) CD300e expression	4 (3%)	0 (0%)	4 (3%)	97 (72%)	52 (79%)	149 (74%)	<0.001
Asynchronous (early) CD35 expression	9 (8%)	4 (15%)	13 (9%)	97 (72%)	47 (71%)	144 (72%)	<0.001
Asynchronous (early) CD14 expression	17 (15%)	2 (3%)	19 (13%)	10 (7%)	2 (3%)	12 (6%)	0.02

Results expressed as number of AML cases and percentage between brackets; p-values correspond to comparisons between * total AML-*NPM1*^{mut} vs. AML-*NPM1*^{wt} patient groups. No statistically significant differences were observed among *FLT3*-ITD+ vs. *FLT3*-ITD- patient groups. APL patients systematically lacked asynchronous monocytic maturation patterns.

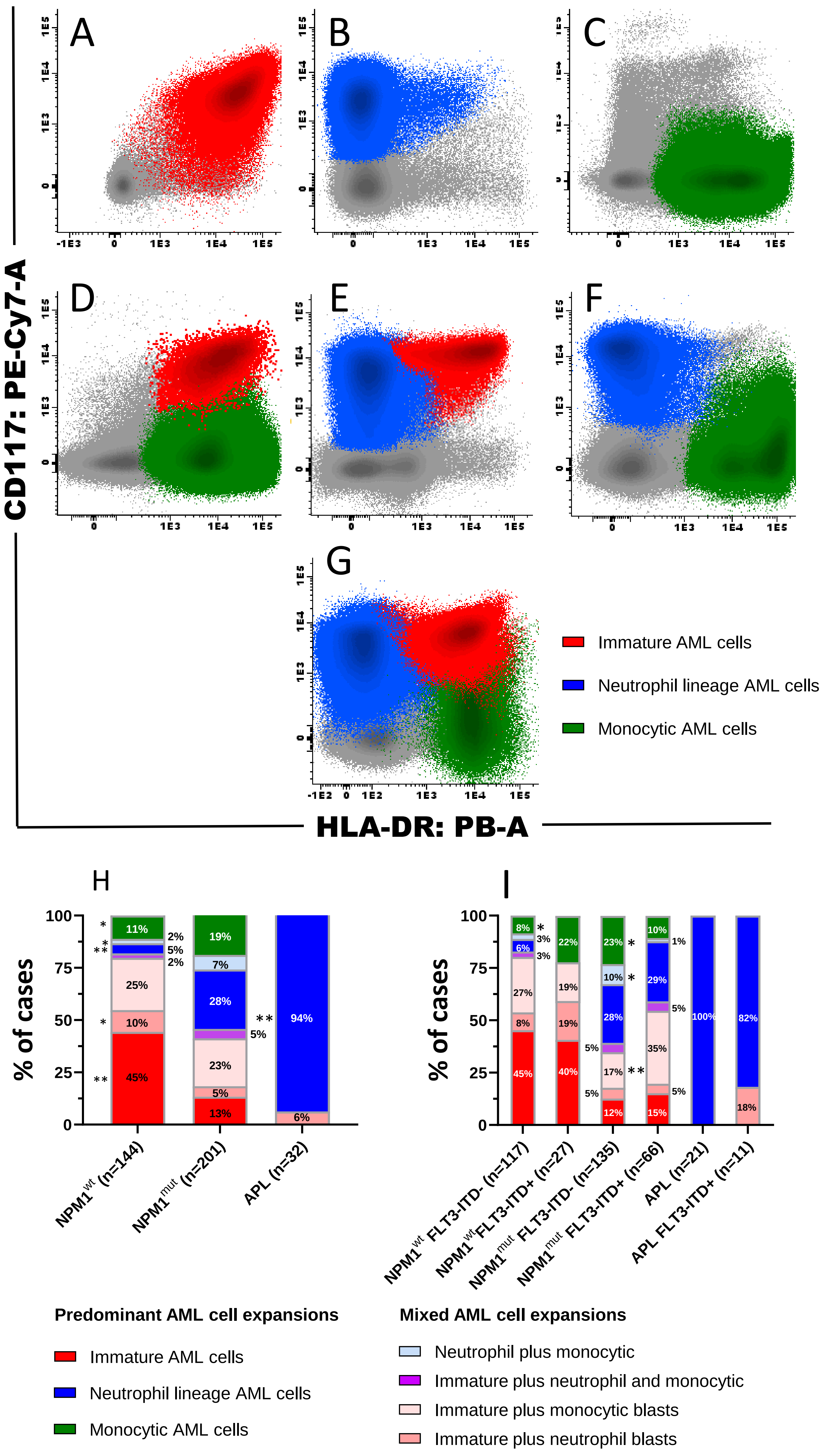
A
B
C
D
E
F
G
CD117: PE-Cy7-A
HLA-DR: PB-A
Immature AML cells
Neutrophil lineage AML cells
Monocytic AML cells
H
I
% of cases
NPM1wt (n=144)
NPM1mut (n=201)
APL (n=32)
NPM1wt FLT3-ITD- (n=117)
NPM1wt FLT3-ITD+ (n=27)
NPM1mut FLT3-ITD- (n=135)
NPM1mut FLT3-ITD+ (n=66)
APL (n=21)
APL FLT3-ITD+ (n=11)
Predominant AML cell expansions
Immature AML cells
Neutrophil lineage AML cells
Monocytic AML cells
Mixed AML cell expansions
Neutrophil plus monocytic
Immature plus neutrophil and monocytic
Immature plus monocytic blasts
Immature plus neutrophil blasts

Supplementary Figure 1. Relative distribution of BM leukemia cell subset profiles in AML and APL patients grouped according to the presence vs. absence of *NPM1* and/or *FLT3*-ITD mutations. Major expansions of a single predominant leukemia cell population (≥80% of all leukemia cells) are illustrated in Panels A-C for $CD117^{+}HLADR^{+}$ immature AML cells, $CD117^{+/het}$ $HLA\text{-}DR^{-}$ neutrophil lineage-, and $CD64^{+/hi}$ $HLA\text{-}DR^{+}$ monocytic lineage-committed leukemia cells (depicted in red, blue and green colors, respectively). Mixed expansions of ≥2 AML cell populations are depicted in Panels D-G. In panels H and I, stacked bars represent the frequency of cases showing one predominant (major) vs. mixed leukemia cell expansions of: i) immature plus neutrophil (dark pink), ii) immature plus monocytic (light pink), iii) neutrophil plus monocytic (light blue) and, iv) immature plus neutrophil and monocytic lineage committed leukemia cells (purple). *, $p<0.05$ and; **, $p<0.03$ vs. *NPM1*^{mut} AML patients (Panel H) and vs. *FLT3*-ITD^{-} cases (Panel I), respectively.

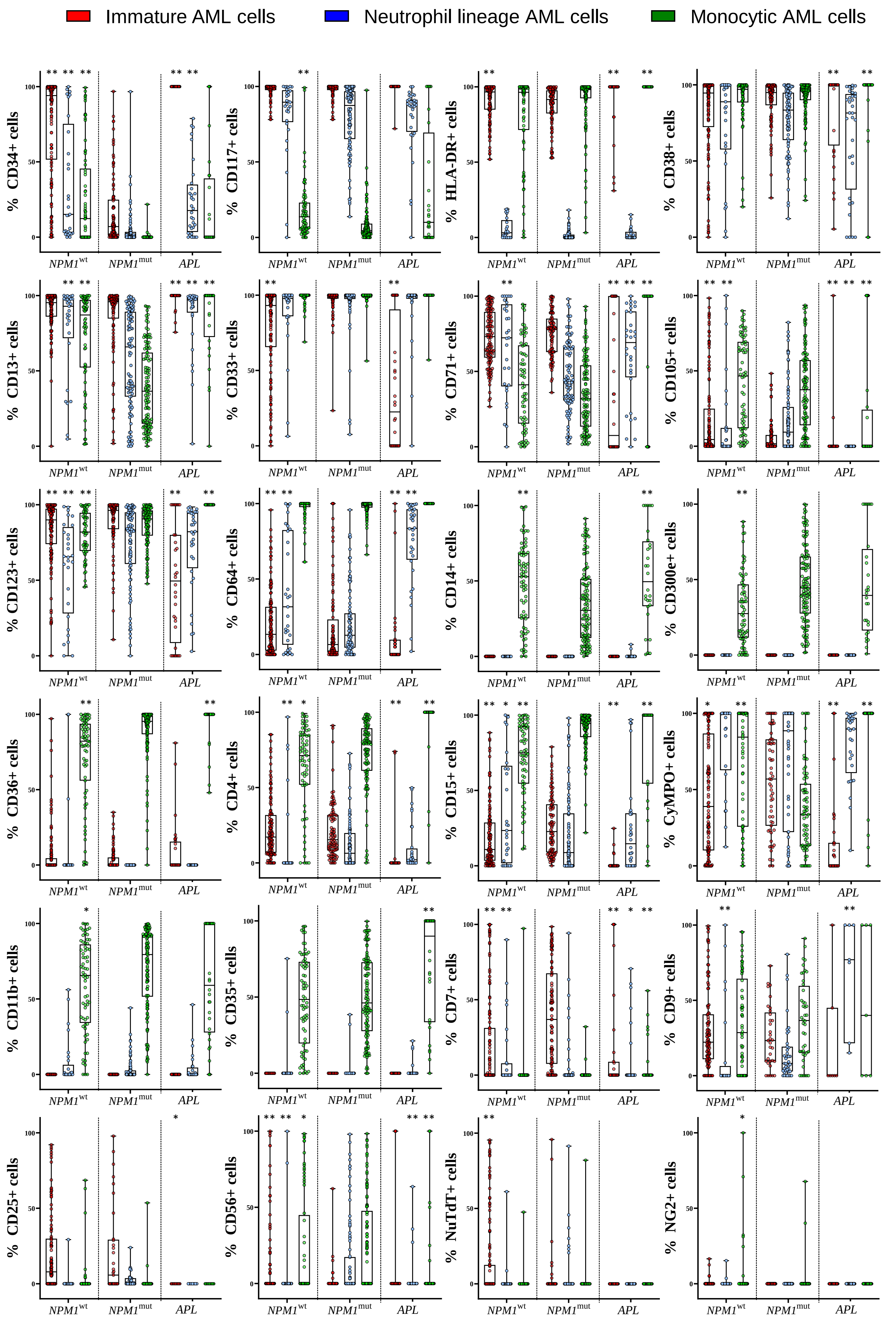

Supplementary Figure 2. Immunophenotypic patterns of distinct subsets of immature CD117^{+}HLADR^{+} (red dots), CD117$^{+/het}$ HLA-DR^{-} neutrophil lineage- (blue dots) and CD64$^{+/hi}$ HLA-DR^{+} monocytic lineage-committed (green dots) BM leukemia cells from patients with AML-*NPM1*^{mut} vs. AML-*NPM1*^{wt} and APL with *PML::RARA* gene rearrangement studied at diagnosis. Results are expressed as percentage of positive cells for individual antigens included in the EuroFlow AML/MDS panel for which statistically significant differences were found among the three patient groups. Notched boxes extend from the 25th to the 75th percentile values; the lines in the middle and vertical lines correspond to median values and minimum and maximum values, respectively. *, p <0.05 and; **, p <0.03 vs. *NPM1*^{mut} AML patients, respectively.

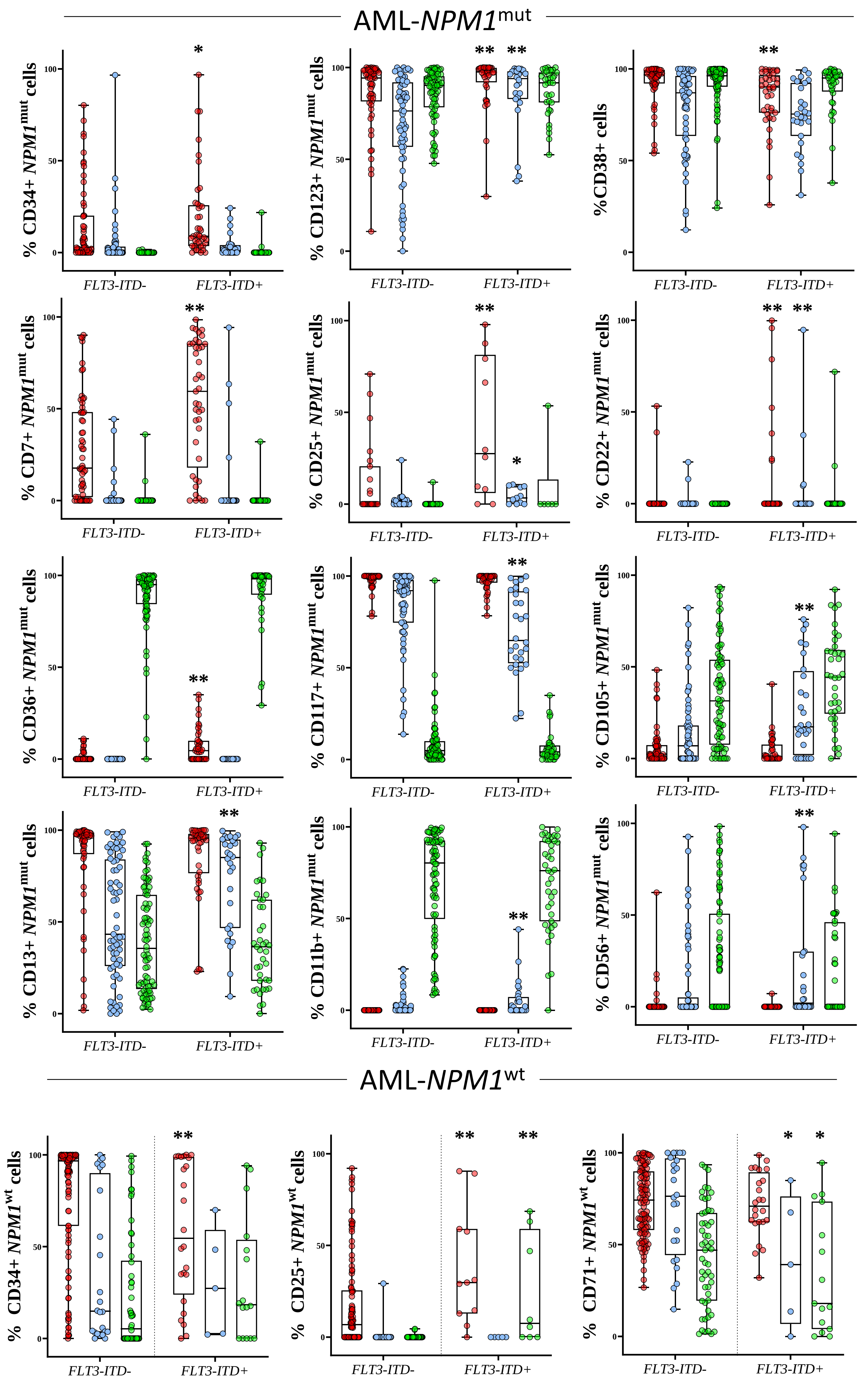

Supplementary Figure 3. Immunophenotypic patterns associated with *FLT3*-ITD among immature CD117$^+$HLADR$^+$ AML cells (red dots), CD117$^{+/het}$ HLA-DR$^-$ neutrophil lineage- (blue dots) and CD64$^{+/hi}$ HLA-DR$^+$ monocytic lineage-committed (green dots) BM leukemia cells from AML patients with *NPM1*^{mut} (upper panels) and *NPM1*^{wt} (lower panels) at diagnosis. Results are expressed as percentage of positive cells for individual antigens from the EuroFlow AML/MDS panel showing statistical differences among patient groups. Notched boxes extend from the 25th to the 75th percentile values; the lines in the middle and vertical lines correspond to median values and minimum and maximum values, respectively. *, p <0.05; **, p <0.03 for comparisons vs. *FLT3*-ITD$^-$ AML patients, respectively.
